# Remote monitoring of automated peritoneal dialysis reduces mortality, adverse events and hospitalizations: a cluster-randomized controlled trial

**DOI:** 10.1093/ndt/gfae188

**Published:** 2024-08-20

**Authors:** Ramón Paniagua, Alfonso Ramos, Marcela Ávila, María-de-Jesús Ventura, Armando Nevarez-Sida, Abdul Rashid Qureshi, Bengt Lindholm, Mexican Nephrology Collaborative Study Group, Diana Pérez-Morán, Miguel A Trejo-Villeda, María D Zavaleta-Diaz, Beatriz Hernández-Franco, Alejandro B Hinojosa-Rojas, Leysy Rosales-Chavarría, María R Romano-Bárcenas, Juana Morales-Monterrosas, Jovita Hijui-Xopa, María-Guadalupe Suárez-López, Belisario Domínguez, Mario Rojas-Díaz, Nancy Ávila-Ortega, Carlos McGregor, Angelica Cruz-Baltazar, Rubén Acosta-Jurado, María Begonia-Ilabaca, Patricia Gómez-Torres, Emilia Cantoral-Farfán, Norberto Ávila-Osorio, Cristina Rodríguez-Esquivel, Lucina Hernández-Cervantes, Fabiola Reyes, Clara V Ramírez-Loera, Maritoña Camarillo, Alejandro Sánchez-Mendoza, Israel Chávez-Palacios, Adrián Ramírez-Cárdenas, Ofelia Galván-Vela, Diana P García-Velásquez, Edith M De-León-Lagunas, Ofelia Sáenz-Flores, David Utrera-Ruiz, María I Rivera-Juárez, María A Soto-Gómez, Margarita Jiménez-Garzón, Marco A Nepomuceno De Florencio, José F Álvarez-Reséndiz, Sandra Rodríguez-Badillo, María E Solís-Gómez, María E Reyes-López-León, María L Romo-Flores, Víctor I Tejeda-González, Laura E Aguilar-Fletes, Samara A Plascencia-Coutiño, Ámbar P Uriarte-Loaiza, A Martha, Padilla-Mercado, Karina Arroyo-Cuevas, Laura M Díaz-Canchola, Laura Quezada-Jauregui, Daniel Gil-Romero, Alma D Cansino-Villagómez, Silverio Lara-Robles, María T Muñoz-Rivera, Rosenda A Zurita-Rodríguez, Teresita J Rodríguez-Vega

**Affiliations:** Unidad de Investigación Médica en Enfermedades Nefrológicas, Hospital de Especialidades, Centro Médico Nacional Siglo XXI, Instituto Mexicano del Seguro Social, Ciudad de México 06720, Mexico; Macrotech, Mexico City, Mexico; Unidad de Investigación Médica en Enfermedades Nefrológicas, Hospital de Especialidades, Centro Médico Nacional Siglo XXI, Instituto Mexicano del Seguro Social, Ciudad de México 06720, Mexico; Unidad de Investigación Médica en Enfermedades Nefrológicas, Hospital de Especialidades, Centro Médico Nacional Siglo XXI, Instituto Mexicano del Seguro Social, Ciudad de México 06720, Mexico; Unidad de Investigación Médica en Enfermedades Nefrológicas, Hospital de Especialidades, Centro Médico Nacional Siglo XXI, Instituto Mexicano del Seguro Social, Ciudad de México 06720, Mexico; Division of Renal Medicine and Baxter Novum, Department of Clinical Science, Intervention and Technology, Karolinska Institutet, Stockholm, Sweden; Division of Renal Medicine and Baxter Novum, Department of Clinical Science, Intervention and Technology, Karolinska Institutet, Stockholm, Sweden

**Keywords:** adverse events, automated peritoneal dialysis, cluster randomized control trial, mortality, remote patient monitoring

## Abstract

**Background:**

Remote monitoring (RM) of patients on automated peritoneal dialysis (APD) prevents complications and improves treatment quality. We analyzed the effect of RM-APD on mortality and complications related to cardiovascular disease, fluid overload and insufficient dialysis efficiency.

**Methods:**

In a cluster-randomized, open-label, controlled trial, 21 hospitals with APD programs were assigned to use either RM-APD (10 hospitals; 403 patients) or conventional APD (11 hospitals; 398 patients) for the treatment of adult patients starting PD. Primary outcomes were time to first event of: (i) Composite Index 1 comprising all-cause mortality, first adverse events and hospitalizations of any cause, and (ii) Composite Index 2 comprising cardiovascular mortality, first adverse event and hospitalizations related to cardiovascular disease, fluid overload and insufficient dialysis efficiency. Secondary outcomes were time to first event of individual components of the two composite indices, and rates of adverse events, hospitalizations, unplanned visits and transfer to hemodialysis. Patients were followed for a median of 9.5 months. Primary outcomes were evaluated by competing risk analysis and restricted mean survival time (RMST) analysis.

**Results:**

While time to reach Composite Index 1 did not differ between the groups, Composite Index 2 was reached earlier (ΔRMST: –0.86 months; *P* = .02), and all-cause mortality [55 vs 33 deaths, *P* = .01; sub-hazard ratio (sHR) 1.69 (95% confidence interval 1.39–2.05), *P* < .001] and hospitalizations of any cause were higher in APD group than in RM-APD as were cardiovascular deaths [24 vs 13 deaths, *P* = .05; sHR 2.44 (95% confidence interval 1.72–3.45), *P* < .001] and rates of adverse events and hospitalizations related to cardiovascular disease, fluid overload or insufficient dialysis efficiency. Dropouts were more common in the APD group (131 vs 110, *P* = .048).

**Conclusions:**

This randomized controlled trial shows that RM may add significant advantages to APD, including improved survival and reduced rate of adverse events and hospitalizations, which can favorably impact the acceptance and adoption of the therapy.

## Graphical Abstract

**Graphical Abstract:**
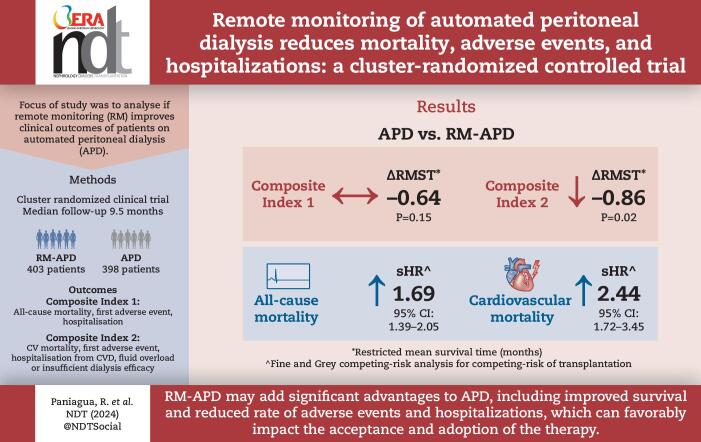

KEY LEARNING POINTS **What was known:**

- A remote monitoring (RM) device incorporated into an automated peritoneal dialysis (APD) cycler has become available in recent years and use of the RM-APD system may overcome some of the inherent limitations of home dialysis therapy by allowing continuous surveillance of peritoneal dialysis (PD) treatment data in patients undergoing RM-APD at home.
- RM-APD allows the clinical staff to follow patients’ adherence to the prescribed PD schedule, ultrafiltration volumes, effective dialysis time, and inflow and outflow patterns of PD solutions and based on this information modify PD prescriptions if needed.
- RM-APD was reported to decrease adverse events, hospitalizations and unscheduled visits; however, the information has been derived from non-randomized trials with short follow-up and often small number of patients, and a randomized controlled trial has been lacking.

**This study adds:**

- Information on the effect of RM-APD on survival, adverse events, hospitalization and unscheduled visits in the setting of a cluster-randomized controlled trial.
- The selected design and analysis using competing risk analysis and restricted mean survival time provide results with increased robustness without the need for assumptions of proportional risk that is required in commonly used survival analysis by Cox regression.
- The observed reductions in mortality and adverse events related to fluid overload and insufficient dialysis efficiency are congruent with the usefulness of information provided by RM-APD, which allows the clinical staff to check adherence to treatment, effective time of dialysis and ultrafiltration.

**Potential impact:**

- RM-APD provides the PD team with a new and powerful tool for improving clinical outcomes in patients treated with APD.
- RM promotes new routines for optimizing the vigilance of care of APD patients through close follow-up of variables related to the pathophysiology of treatment-related adverse events and mortality.
- Improved clinical outcomes with reduction of adverse events and less need of hospitalizations can favorably impact the acceptance and adoption of the therapy.

## INTRODUCTION

Peritoneal dialysis (PD) is a long-term viable kidney replacement therapy [1]. Its use varies widely around the world, from close to zero in some regions of France to >50% of all dialysis patients in some regions of Mexico, and 69% in Hong Kong [2, 3].

Mortality in PD is similar or even lower than in hemodialysis (HD) [4–6]. While PD and home HD are both methods for self-care dialysis treatment that grant the patients more autonomy to carry out their usual daily activities compared with in-center HD, PD is technically simpler than HD. These advantages may be even more significant with automated PD (APD), which is usually performed at night [7–10].

Recent incorporation of electronic data transfer technology into the APD equipment [11] helps the healthcare team to make timely adjustments of therapy prescriptions [12, 13] by providing immediate access to information gathered by remote monitoring (RM) of treatment data cycle-by-cycle, day-by-day on the evolution of important indicators of dialysis efficiency such as ultrafiltration, the effective time of dialysis and the adherence to prescribed treatment [14]. By proactively detecting problems and specific needs for patient training, the use of RM may reduce the number of complications, unscheduled visits to the hospital, and the incidence and duration of hospitalization [13–15], allowing the kidney care team to optimize organization, improve the efficiency of care and reduce the costs of therapy [16, 17].

The current knowledge about the effects of RM in APD has been derived from observational studies or cohort surveillance studies without intervention and has not been validated in the context of controlled clinical trials. Furthermore, survival and incidence of preventable complications have not been studied in such trials. The objective of this study was to assess the effect of using RM in APD (RM-APD) on mortality and complications related to fluid overload and effective hours of dialysis through a randomized controlled clinical trial.

## MATERIALS AND METHODS

### Design

A cluster-randomized, open-label, multicenter controlled clinical trial with two parallel groups was conducted to compare conventional APD (APD group) vs RM-APD (RM-APD group). The cluster design prevents the possible bias of doctors and patients due to perception of anticipated advantage in the intervention group [18]. The follow-up time was at least 1 year from the inclusion of the last patient. The study was approved by the National Ethics and Research Committees of the Mexican Social Security Institute (R-2018-785-035, 3 May 2018), and by the Local Committees of each hospital. The study was conducted in accordance with the provisions of the Declaration of Helsinki and Tokyo with the amendments in Venice (1983) and was registered in ClinicalTrials.gov (NCT04034966).

### Hospitals

Hospitals with PD programs comprising at least 100 prevalent and 50 incident PD patients per year, under the supervision of experienced nephrologists and nurses, were included in the study. The characteristics and clinical settings of the institutions and hospital networks participating in the study are described in the Supplementary data.

### Patients

Adult (>18 years) men and women under 70 years of age with kidney failure and onset of PD within 3 months were included. No selection was made for the disease of origin of chronic kidney disease. Patients were enrolled from 1 August 2018 to 30 August 2019. None of them was previously using RM-APD since it was introduced in Instituto Mexicano del Seguro Social (IMSS) hospitals for the purpose of the study; two non-IMSS hospitals had short previous experience of RM-APD in other patients.

Patients with positive serology for HIV, hepatitis B or C, with cancer, under treatment with immunosuppressant, possibility of kidney transplantation in a short time, and patients with acute complications in the 30 days prior to recruitment were excluded. Patients gave their signed informed consent without previous knowledge of the assigned group of the hospital.

### Intervention

The APD equipment was the same in both groups (Homechoice Claria^®^, Baxter, S.A. de C.V.), and was equipped with device for RM (Sharesource^®^ connectivity platform, Baxter Healthcare, Deerfield, IL, USA) with modem to transmit the information recorded. In the RM-APD group, the PD team had access to all information recorded since their inclusion in the study. In the APD group, the PD team did not have access to the information transmitted by the RM device; the management of patients in the APD group was carried out in the usual way. The study coordinating team always had access to the information generated by the RM devices of the two groups and thus was able to collect the data but did not participate in clinical decisions. In both groups, the treating physician was responsible for all PD prescriptions, and routines and management for visits of patients were performed according to the protocols of each hospital. Doctors and nurses received training in the management of the RM-APD platform before the enrollment through interactive workshops, and all were provided with personal computers and smartphones for communication with patients and the study coordinating center. The training was reinforced with additional workshops at the third and sixth month after the beginning of the study. At any time, online and/or in person, individualized assistance was provided upon request. The thematic content of courses is described in Supplementary data. All patients were free to go to the hospital for any doubt about the handling of dialysis devices as well as for any health problem related or not to PD. All patients received training in the management of PD through interactive workshops. The study coordinating team defined the flag rule settings in the device. The setting points are shown in Supplementary data, Table S1. The algorithm for routine analysis is shown in Supplementary data, Fig. S1.

### Randomization

Randomization was carried out at the cluster level, that is, each hospital was considered as a unit and all the patients in that unit were included in the same group. Group allocation was done by simple 1:1 randomization with a table of random numbers. Candidate patients were randomly selected (using table of random numbers applied to the last two digits of the social security number) from the lists of patients in the APD clinics of the participating hospitals.

### Outcomes

Composite indices were constructed based on four SONG-PD (Standardized Outcomes in Nephrology-Peritoneal Dialysis) core outcomes: mortality, cardiovascular diseases, PD infection and technique survival, complemented by SONG-PD middle and outer tiers outcomes as appropriate (see below) such as cardiovascular disease with fluid overload, hypertensive crisis, stroke and other [18].

The primary outcomes were time to the first event of Composite Index 1 that included mortality, first adverse events, and first hospitalizations of any cause, and of Composite Index 2 which included (i) cardiovascular mortality, first adverse event and first hospitalization due to cardiovascular or ultrafiltration-associated events (heart failure, cardiovascular dysautonomia, acute myocardial infarct, syncope, edema, stroke, pleural effusion, angina, hypertensive crisis and sudden death) and (ii) mortality and events that could be attributed to ineffective dialysis (uremic manifestations, such as malnutrition, acidosis, hyperkalemia and other electrolyte disorders).

Secondary outcomes were the time to the first event of the individual components of the two composite indices: all-cause and cardiovascular disease–related deaths, adverse events and hospitalizations, respectively. Furthermore, rates of adverse events, hospitalizations linked to adverse events possibly related to potentially preventable causes such as fluid overload (edema, hypertension and heart failure), unplanned visits and technique failure were also analyzed.

Dropouts included all causes of patients terminating their participation: by patient decision (leaving the study, change of dialysis modality, lack of caregiver), for administrative reasons (change of residence, loss of validity in social security), kidney transplant, medical causes (change of dialysis modality, surgery, accident) and deaths.

### Data acquisition

Demographic data and relevant data concerning the underlying kidney disease and comorbidity at baseline were obtained from the clinical records. Information concerning adverse events and hospitalizations was recorded from each hospital and confirmed by interviews with patients or their caregivers. Doctors and nurses were instructed to review daily the data provided by the RM-APD device such as: the day and time of connection, the duration of treatment per day, total exchange volume, the number of cycles, use or not of wet day (i.e. daytime exchange in the preceding day), dialysate volume infused, total dwell time, dwell time per exchange, actual time for filling and draining per exchange, drain volume per dwell and total treatment, cycle profile, total and dwell ultrafiltration, and number of events per treatment. The number and reason for alarms, events and bypass drain were also recorded and reviewed (described in the Supplementary data). The RM-APD device requested that patients provided daily input of data on body weight and blood pressure.

### Statistical analysis

Variables are presented as percentages or means with corresponding standard deviations. Demographic and clinical data were compared between the two groups at the onset of the study.

The study employed a cluster-randomized trial design with the hospital as the unit of randomization. Dichotomous outcomes were compared using an adjusted two-sample *t*-test. Generalized estimating equations (GEEs) were utilized to account for individual- and cluster-level covariates and address within-cluster correlation, producing the population average effect of the intervention [19, 20]. Confidence intervals (CIs) were not adjusted for multiple testing.

To control differences in the baseline rate of PD use, a differences-in-differences approach was employed [21]. GEE models were constructed to compare changes over time, incorporating the interaction between groups (RM-APD and APD) and hospitals. Log-binomial and log-Poisson models were used for categorical outcomes, while GEE models with normal distribution and identity link were applied for continuous models [21].

Statistical significance was set at *P* < .05. Group comparisons included the non-parametric Wilcoxon test for skewed continuous variables, Student's *t*-test for normally distributed variables and Fisher’s exact test for nominal variables.

Survival in the two groups was compared adjusting for age, sex and center (hospitals) using: (i) competing risk analysis according to Fine and Gray models [22–24] taking kidney transplantation as a competing risk to establish cumulative incidence curves and sub-hazard ratios (sHRs) with 95% CIs [22–24]; and (ii) restricted mean survival time (RMST) models with RMST representing the area under the survival curve up to a specific truncation time point, providing a reliable quantitative survival estimate (expressed as ∆RMST indicating gain or loss of number of months alive in one group as compared with the other group) [25]. The secondary outcomes were assessed using Cox proportional hazards, expressing results as HRs with 95% CI.

All statistical analyses were conducted using Stata 18.0 (Stata Corporation, College Station, TX, USA) and SAS 9.4 level 1 M8 (SAS Campus Drive, Cary, NC, USA).

## RESULTS

Twenty-one hospitals were included and randomly assigned to receive treatment with RM-APD (10 hospitals; 403 patients) or conventional APD (11 hospitals; 398 patients). The recruitment of participants is shown in Supplementary data, Fig. S2. The two groups were similar both in terms of baseline characteristics of the participating hospitals (Table 1) and baseline characteristics of the patients (Table 2); however, baseline diagnoses of hypertension and peripheral vascular disease were more common and circulating concentrations of calcium were lower in the APD group. Some variables tended to be different between the two groups, but differences did not reach statistical significance; for details see Supplementary data, and the Discussion section. The patients were followed for median 9.5 (interquartile range 3.2–14) months.

**Table 1:** Characteristics of participating hospitals in cluster group RM-APD and cluster group APD. Number of patients, healthcare professionals and training sessions or hours.

	RM-APD	APD	*P*
Patients in pre-dialysis clinic	275 ± 328	259 ± 419	.848
CAPD patients (*n*/hospital)	203 ± 229	219 ± 118	.09
APD patients (*n*/hospital)	198 ± 84	167 ± 102	.47
HD patients (*n*/hospital)	250 ± 190	336 ± 16	.41
New patients on PD (*n*/month)	10.5 ± 4.2	10.1 ± 5.0	.81
Nephrologists (*n*/hospital)	3.20 ± 3.25	2.88 ± 2.13	.77
Nurses in nephrology ward (*n*/hospital)	5.60 ± 2.80	6.80 ± 3.07	.273
Training sessions in PD	5.10 ± 0.20	4.80 ± 0.60	.457
Training hours by session	3.50 ± 1.10	3.60 ± 1.10	.727
Patients in each training session (*n*/session)	10 ± 8.8	7.00 ± 4.1	.251

Data are expressed as mean ± standard deviation.

CAPD, continuous ambulatory PD.

**Table 2:** Demographics, clinical data and baseline biochemical parameters of patients at baseline.

		Total	APD	RM-APD		
	*n*	*N* = 801	*N* = 398	*N* = 403	*P*-value	ICC
Age (years)	801	50.5 (15.4)	51.5 (15.1)	49.5 (15.7)	.062	0.137
Sex, men, *n* (%)	801	522 (65.2)	262 (65.8)	260 (64.5)	.71	0.016
Weight (kg)	801	69.0 (13.7)	69.0 (14.0)	68.9 (13.3)	.96	0.008
Height (cm)	801	163 (8.9)	163(9.0)	163 (8.8)	.76	0.02
Blood pressure systolic (mmHg)	801	132 (18.3)	133 (17.5)	131(19.0)	.14	0.076
Blood pressure diastolic (mmHg)	801	79.9 (12.1)	80.2 (11.5)	79.7 (12.6)	.52	0.029
Urine volume/24 h (mL)	210	897 (461)	840 (354)	947 (535)	.095	0.693
Underlying kidney disease, *n* (%)						
Obstructive uropathy	801	6 (0.7)	2 (0.5)	4 (1.0)	.69	0.044
PCKD	801	8 (1.0)	4 (1.0)	4 (1.0)	1.00	0.019
Diabetic nephropathy	801	455 (56.8)	223 (56.0)	232 (57.6)	.67	0.123
Hypertension	801	229 (28.6)	133 (33.4)	96 (23.8)	.003	0.241
Unknown	801	137 (17.1)	70 (17.6)	67 (16.6)	.78	0.293
Cardiovascular disease, *n* (%)						
Chronic heart disease	801	5 (0.6)	4 (1.0)	1 (0.2)	.21	0.0003
Ischemic cardiopathy	801	12 (1.5)	7 (1.8)	5 (1.2)	.58	0.003
AMI	801	6 (0.7)	4 (1.0)	2 (0.5)	.45	0.0003
Arrhythmia	801	3 (0.4)	2 (0.5)	1 (0.2)	.62	0.0001
Pericarditis	801	2 (0.2)	2 (0.5)	0 (0.0)	.25	0.765
Stroke	801	8 (1.0)	6 (1.5)	2 (0.5)	.18	0.029
Peripheral vascular	801	10 (1.2)	10 (2.5)	0 (0.0)	<.001	0.709
Hypertension	801	598 (74.7)	320 (80.4)	278 (69.0)	<.001	0.241
Blood biochemistry						
Serum albumin, g/dL	801	3.3 (0.6)	3.3 (0.6)	3.3 (0.6)	.29	0.078
Serum Na, mmol/L	636	136.7 (5.0)	136.7 (4.4)	136.7 (5.6)	.97	0.024
Hb, g/L	760	10.2 (2.1)	10.0 (2.0)	10.4 (2.2)	.008	0.038
Creatinine, mg/dL	801	9.5 (3.9)	9.7 (4.1)	9.3 (3.6)	.18	0.026
Ca, mg/dL	801	8.4 (0.8)	8.3 (0.8)	8.5 (0.8)	<.001	0.027
Phos, mg/dL	801	5.2 (1.5)	5.1 (1.5)	5.2 (1.4)	.42	0.095
Ca × Phos product	801	43.1 (12.4)	42.1 (11.9)	44.2 (12.8)	.018	0.029

Continuous variables are presented as mean (standard deviation). Categorical variables are presented as number (percentage). ICC is adjusted for groups and centers variable.

ICC, Intraclass Correlation Coefficient; PCKD, polycystic kidney disease; AMI, acute myocardial infarction; Na, sodium; Hb, hemoglobin; Ca, calcium; Phos, phosphorus.

### Primary outcome

**Composite index 1**

**Figure 1:**
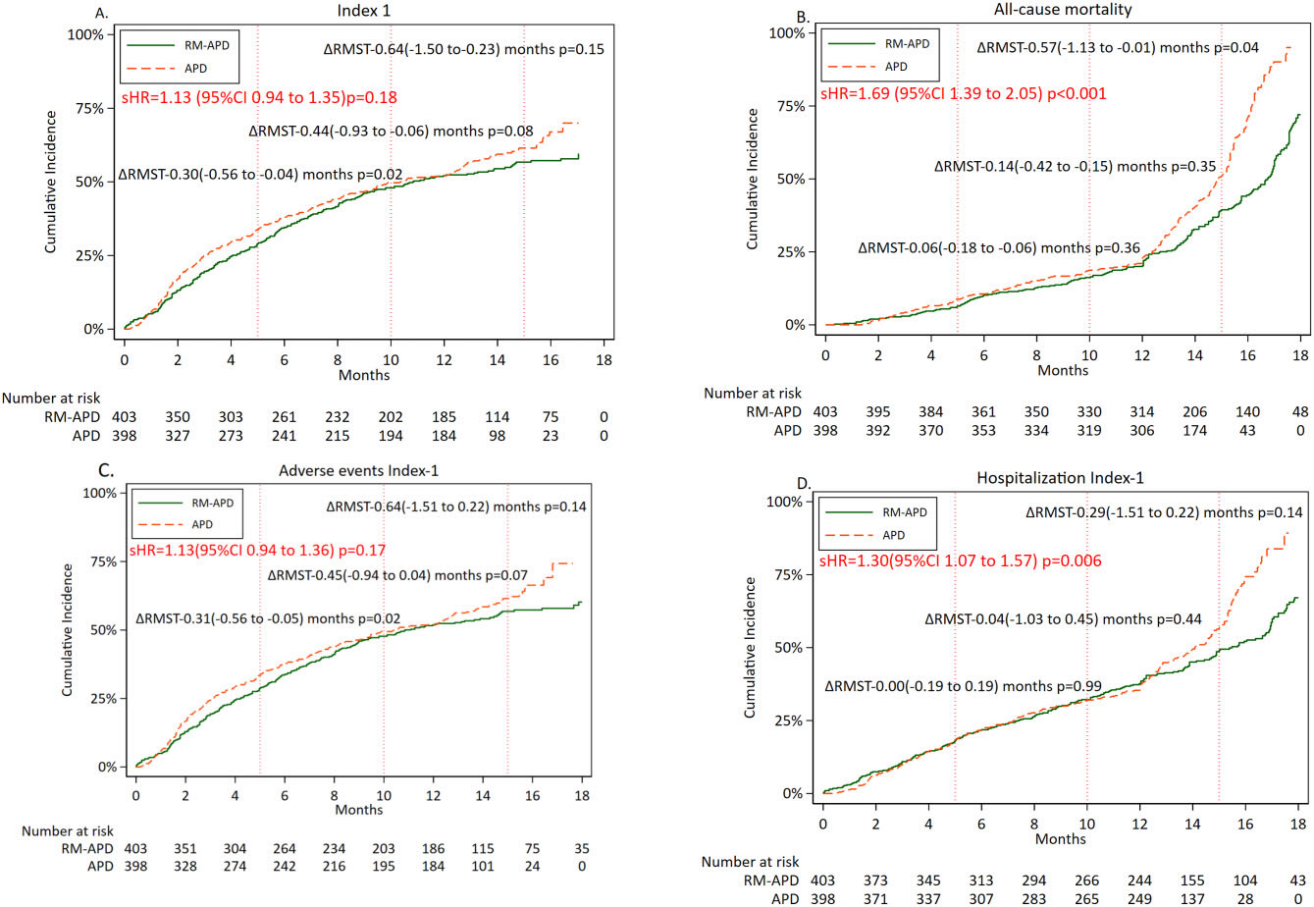
Cumulative incidence rates of Composite Index 1. (**A**) First event of either all-cause mortality, adverse events and hospitalization of any cause; and the first event of the three components of index 1, all-cause mortality (**B**), adverse events (**C**) and hospitalization of any cause (**D**) for patients receiving APD and RM-APD, respectively. Inserts show results of competing risk analysis with sHR and 95% CI for these outcomes in patients using APD and RM-APD, respectively, after adjusting for age, sex and center. Results of RMST analysis with RM-APD as compared with APD at 5, 10 and 15 months for are shown as ΔRMST (95% CI) months.

Cumulative incidence rates of Composite Index 1 and its three components, all-cause mortality, adverse events and hospitalization of any cause, results of competing risk analysis with sHRs with 95% CI, and results of RMST analysis, for each of these four outcomes, for APD versus RM-APD, are shown in Fig. 1A–D. The risk of early occurrence of Composite Index 1 and adverse events were similar in APD and RM-APD; however, all-cause mortality was higher [sHR 1.69 (95% CI 1.39–2.05), *P* < 0.001; Fig. 1A], and hospitalizations of any cause occurred earlier with APD compared with RM-APD.

RMST analysis (Fig. 1A–D) showed that at 15 months, the time to reach Composite Index 1 did not differ between the two groups, nor did time to first adverse event or hospitalization; however, all-cause mortality occurred later in the RM-APD group [ΔRMST at 15 months: –0.57 (95% CI –1.13 to –0.01) months; *P* = .04].

Sensitivity analysis using Cox regression analysis further supported these findings (Supplementary data, Fig. S5A–D).

#### Composite index 2

**Figure 2:**
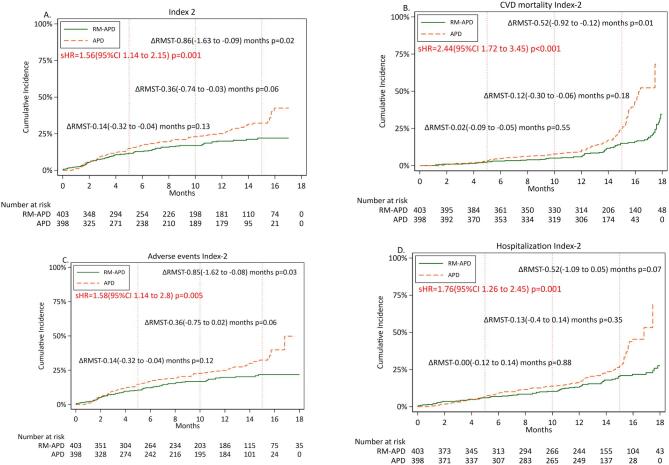
Cumulative incidence rates of Composite Index 2. (**A**) First event of either cardiovascular mortality, or adverse events and hospitalizations caused by cardiovascular events for patients receiving APD and RM-APD, respectively; and the first event of the components of Composite Index 2, cardiovascular mortality (**B**), adverse events (**C**) and hospitalizations (**D**) of cardiovascular origin. Inserts show results of competing risk analysis with sHR and 95% CI for these outcomes in patients using APD and RM-APD, respectively, after adjusting for age, sex and center. Results of RMST analysis with RM-APD as compared with APD at 5, 10 and 15 months are shown as ΔRMST (95% CI) months.

Cumulative incidence rates of Composite Index 2 and its three components, cardiovascular mortality, and adverse events and hospitalization of cardiovascular origin, results of competing risk analysis with sHR (95% CI) and results of RMST analysis for each of these outcomes for APD versus RM-APD, are shown in Fig. 2A–D. The risk of early occurrence of Composite Index 2 and its components, cardiovascular mortality [sHR 2.44 (95% CI 1.72–3.45), *P* < .001; Fig. 2B], adverse events and hospitalizations of cardiovascular origin or related to fluid overload or inefficient dialysis, were all significantly higher with APD as compared with RM-APD.

RMST analysis (Fig. 2A–D) showed that at 15 months, time to reach Composite Index 2 occurred significantly later with RM-APD [ΔRMST –0.86 (95% CI –1.63 to –0.09) months, *P* = .02]. Among individual components of Index 2, cardiovascular mortality (*P* = .01) and time to first adverse event (*P* = .03) also occurred later in RM-APD group, whereas time to first hospitalization (*P* = .07) did not differ between the groups.

Sensitivity analysis using Cox regression analysis further supported these findings (Supplementary data, Fig. S6A–D).

Adverse events were analyzed as rates at each center and then the centers were compared between groups. Table 3 shows the list of events by overall event types and their rates measured as incidence rate ratios (IRRs) in the APD group as compared with the RM-APD group. The RM-APD group displayed higher number and rates (measured as IRR) of mechanical events causing catheter dysfunction, and hospitalizations due to such events; otherwise RM-APD associated with lower number and rates of other types of adverse events including those linked to fluid balance, hospitalizations due to fluid overload or insufficient effectiveness of dialysis, cardiovascular events, “metabolic events” (see explanation in Table 3), and rates of all-cause deaths and cardiovascular deaths were also lower as compared with the APD group. The number and rates of PD infection events (including peritonitis and exit site infections) did not differ between the groups. The peritonitis rate in the studied population was 0.17 (or 1 episode/72 months) in the whole group of patients, and 0.15 (or 1 episode/82 months) and 0.19 (or 1 episode/63.5 months) in RM-APD and APD groups, respectively.

**Table 3:** Number of adverse events and the IRR for events in group APD versus events in group RM-APD, adjusting for patients’ identification and centers.

Type of event	Crude APD/RM-APD	IRR (95% CI)	*P* > |z|
PD infection events	100/86 (*P* = .12)	1.28 (0.69–2.37)	.42
**Mechanical events**	**33/62 (*P* = .001)**	**0.42 (0.18–0.98)**	**.04**
Intra-abdominal pressure events	6/13 (*P* = .07)	0.48 (0.16–1.40)	.18
**Fluid balance events**	**123/35 (*P* = .001)**	**3.44 (1.95–6.07)**	**.001**
**Cardiovascular events**	**79/44 (*P* = .02)**	**1.81 (1.03–3.09)**	**.03**
**Metabolic events**	**108/44 (*P* = .001)**	**2.78 (1.51–5.38)**	**.005**
Other events	53/77 (*P* = .005)	1.01 (0.45–2.29)	.97
**Mechanical hospitalization events**	**18/45 (*P* = .004)**	**0.41 (0.22–0.66)**	**.001**
**FOL and IDE hospitalizations**	**19/7 (*P* = .01)**	**3.24 (1.24–7.53)**	**.03**
**All-cause deaths**	**55/33 (*P* = .01)**	**1.82 (1.18–2.81)**	**.006**
**Cardiovascular deaths**	**24/13 (*P* = .05)**	**2.03 (1.03–3.97)**	**.04**

Data are expressed as mean ± standard deviation.

Bold font indicates significant difference between APD and RM-APD.

Crude APD/RM-APD = crude analysis of number of adverse events with APD vs RM-APD is calculated with Chi-square test. IRR is calculated with zero-inflated Poisson regression model with Bayesian method. IRR adjusts for patients’ identification and centers.

PD infection events: sum of exit site infection + tunnel infection + peritonitis.

Mechanical events: sum of kinking + disconnection + catheter removal + sleeve extrusion (bearing extortion).

Intra-abdominal pressure events: sum of abdominal discomfort + peritoneal/pleural fistula + intra-abdominal pressure + leakage + hemoperitoneum + hernia.

Fluid balance events: sum of pleural effusion + dehydration + overhydration.

Cardiovascular events: sum of unstable angina + hypertensive crisis + stroke + heart failure + hypotension + acute myocardial infarct + congestive heart failure + chronic heart failure + convulsion + syncope + sudden death.

Metabolic events: sum of malnutrition + hyperglycemia + hypoglycemia + hyperkalemia + hyponatremia + diabetic foot + acid base disorder + uremia.

Other events: sum of gastrointestinal disorders + urinary tract infection + respiratory tract infection.

FOL and IDE hospitalizations: hospitalizations due to fluid overload (FOL) and insufficient dialysis efficiency (IDE).

The dropout rates were slightly but significantly higher in the APD group (Table 4). The rate of patients transferred to HD was however similar in the two groups. When causes of PD discontinuation (adherences, peritonitis, resistant exit site infection, ultrafiltration failure, abdominal surgery and catheter dysfunction) were disaggregated, none of the specific causes was significantly different between APD and RM-APD. The RM-APD group showed a trend towards higher rates of telephone consultations while visits to medical office and emergency room did not differ significantly between the two groups.

**Table 4:** Number and rates of dropouts and unscheduled visits.

	RM-APD		APD		
		Events rates		Events rates	*P*
**Dropouts, total**	**110**	**23.97 ± 7.52**	**131**	**31.71 ± 8.35**	**0.048**
Shift to HD	42	9.42 ± 4.50	36	9.21 ± 4.80	.970
Peritonitis	16	3.9 ± 3.4	21	5.2 ± 3.8	.42
UF insufficiency	11	2.4 ± 2.7	5	1.0 ± 2.2	.22
Other causes	12	3.0 ± 3.1	6	1.9 ± 3.1	.44
Surgery	6	1.4 ± 2.2	7	1.7 ± 2.2	.77
Kidney transplant	7	3.23 ± 1.50	12	5.96 ± 4.88	.45
** CVD deaths**	**13**	**2.9 ± 2.5**	**24**	**6.2 ± 3.8**	**0.032**
** All cause deaths**	**33**	**7.4 ± 2.0**	**55**	**13.5 ± 5.8**	**0.006**
Unscheduled visits, total	234	46.23 ± 41.06	282	71.24 ± 63.14	.324
Telephone	23	5.99 ± 7.93	4	0.94 ± 1.32	.063
Medical office	141	28.06 ± 29.25	184	48.68 ± 50.44	.295
Emergency room	92	17.98 ± 16.54	97	22.40 ± 21.76	.629

Data are expressed as mean ± standard deviation.

Bold font indicates significant difference between APD and RM-APD.

Rates were calculated for each center as: (number of unscheduled visits × 1200)/(total months of follow-up), this normalized the visits to 100 patients/year at risk at center. Dropouts included all causes of patients terminating their participation: by patient decision (leaving the study, change of dialysis modality, lack of caregivers), for administrative reasons (change of residence, loss of validity in social security), kidney transplant (*n* = 19; RM-APD *n* = 7 and APD *n* = 12), medical causes (change of dialysis modality, surgery, accident) and deaths (*n* = see Table 3).

UF, ultrafiltration; CVD, cardiovascular disease.

Number of hospitalizations and the IRR for hospitalizations in APD versus RM-APD are shown in Supplementary data, Table S2.

## DISCUSSION

Previous studies have shown advantages of RM-APD over conventional APD [26–28] including improved technique survival. However, the influence of RM-APD on specific causes of morbidity and mortality has not been analyzed in a randomized control trial previously. The data presented here—obtained in the first randomized controlled trial addressing this topic—show that RM-APD compared with conventional APD was associated with lower all-cause and cardiovascular mortality as well as longer time to reach Composite Index 2 (i.e. an index comprising time to first cardiovascular death, adverse event or hospitalization of cardiovascular origin or associated with ultrafiltration or inefficient dialysis). Furthermore, similar differences between RM-APD and APD were found in the same variables when their incidence rates during the whole follow-up period were analyzed.

The positive impact of RM-APD on events captured by Composite Index 2 is important considering that cardiovascular diseases are the most frequent causes of mortality and comorbidity in patients undergoing kidney replacement therapy [29, 30]. Fluid overload is a frequent complication of PD [31–33] that is determined by sodium and water intake, residual kidney function and achieved peritoneal ultrafiltration volumes, and could be linked to inflammation, abnormal remodeling of left ventricle, diastolic dysfunction, major adverse cardiovascular events and hospitalizations [32, 34–3,8]. In this study, the two groups had similar urine volumes and serum albumin levels, and no specific intervention was prescribed regarding fluid intake or diet; thus, close surveillance of ultrafiltration by RM—and when needed followed by changes in prescriptions based on this information—appears to be a unique factor, possibly positively affecting hemodynamic variables, and fluid overload [14, 39]. Longer dwell time of dialysis fluid in the peritoneal cavity may increase peritoneal clearances of small and middle-sized molecules [39]. RM-APD facilitates timely adjustments of hours of treatment, number of exchanges and dwell volumes for optimization of dialysis efficiency [11]. Furthermore, RM increases adherence to treatment [13, 14]. It is likely that these factors acting together may contribute to explaining at least in part the observed benefits of RM-APD on Composite Index 2 and its components.

Our results are supported by the findings of the RMST analysis showing small but significant differences in favor of RM-APD. Estimates of relative risk such as sHR and HR do not provide a quantitative measure of differences in survival time or survival rate and can be very high even if the absolute effect of factors on survival time is small; therefore, these measures need to be considered together with other indicators, such as median survival time or survival rate at a given time point, to understand their clinical implications. RMST analysis, on the other hand, provides an absolute measure of implications for survival time, such as differences in survival time, for different groups at a given time point [25].

RM-APD allows early detection of alterations in the pattern of the inflow and outflow rate of PD solutions, which may not always be noticed by the patient. The possibility of having objective evaluations could explain why mechanical complications were diagnosed more frequently in the RM-APD group whereas the number and rates of several other types of adverse events (Table 3) were significantly lower among patients receiving RM-APD. There were no differences in rates of PD-related infections between the two groups. This is expected since RM-APD does not address events involved in the etiology of infections or in the infection prevention process.

RM-APD should allow closer follow-up with a better ability to detect problems such as insufficient ultrafiltration, thereby facilitating appropriate actions addressing these problems with timely decisions to transfer those patients not responding to HD, and lower frequency of technique failure has been described with RM-APD as compared with APD [40]. In this study, the rate of dropouts was slightly lower but shifts to HD were not significantly more frequent in the RM-APD group (Table 4).

Differences between APD and RM-APD in Composite Index 1 and 2 and their components became more evident in the second half of follow-up. We do not have a specific explanation for this time lag, except that in the RM-APD group, the transfer to HD and dropouts due to causes such as accidents and non-PD-related infections were more common and occurred earlier. Deaths from arrhythmia and stroke also occurred more often and earlier. It is possible that this influenced the results in the first 12 months. The results suggest non-proportional risk, and for this reason we performed analysis of RMST [40] for all outcomes (see Figs 1A–D and 2A–D), whereas results of Cox model regression analysis are presented in Supplementary data, Figs S5 and S6).

Our results indicate that in the setting of a randomized control trial, clinical outcomes of patients treated by PD may improve in patients using RM-APD, but it is obvious that these results can only be obtained if the RM-APD tool is used adequately. While RM-APD makes it possible to monitor therapeutic compliance, effective dialysis time, ultrafiltration volumes, and inflow and outflow patterns of dialysis fluid, and actions of patients in response to alarms indicating deviations from targets set by the nephrologist [41], the clinical usefulness of RM-APD is critically dependent on how this tool is being used by physicians and nurses. Several algorithms for routine review have been suggested [ 42, 43]. It is important to mention that RM is well accepted by both healthcare teams and patients, as was recently reported [41].

Among major strengths of this study are its design, aiming at limiting the potential bias of selection due to the possible perception of anticipated advantage in the intervention group, and the inclusion of an adequate number of centers, and patients, as well as the confirmation of results using RMST which is a more robust statistical analysis that does not require the assumptions of the Cox proportional hazards model. The study also has some limitations that should be considered. There are potential pitfalls associated with the use of a cluster-randomized design that may have an impact on the results [44]. While none of the registered characteristics of participating centers differed between the two groups (Table 1), the proportion of patients with a diagnosis of hypertension and peripheral vascular disease was higher and the circulating concentration of calcium was lower in the APD group (Table 2); it is possible that these differences may reflect differences between centers as regards routines and methods for diagnoses and laboratory measurements, respectively. The actual systolic and diastolic blood pressure at the start of the study did not differ between the two groups (Table 2). Age tended to be higher and urine volume/24 h tended to be lower among patients in the APD cohort; however, age distribution and number of patients with significant urinary volumes (≥100 mL/24 h) was similar in the two groups (Supplementary data, Figs S3 and S4, respectively). As mentioned above, the generalizability of the results is influenced by the setting of the trial and if the RM-APD tool is used adequately; extrapolations may not be valid for other settings and patient populations with different characteristics. The socioeconomic conditions differ among countries. For example, the Mexican population is younger than those in developed countries; median age is 29 years and 5% are older than 65 years. Furthermore, PD centers are large; the mean number of PD patients is close to 150 per center for the 250 hospitals of the IMSS. Diabetes, anemia and hypoalbuminemia were frequent among the patients included in the present study; these characteristics could have had an impact on mortality and comorbidity despite similar characteristics at baseline in the two groups.

In summary, this randomized controlled trial shows that in patients with APD the use of RM delayed the occurrence of adverse events and hospitalizations linked to cardiovascular disease, fluid overload and insufficient dialysis, as well as all-cause mortality and mortality of cardiovascular origin. Mortality rates, adverse events and hospitalizations, as well as days of hospitalization and unscheduled visits were also reduced with the use of RM-APD.

In conclusion, the use of RM of patients undergoing automated PD adds important advantages including improved survival, lower number of adverse events and hospitalizations, which can favorably impact the acceptance and adoption of the therapy.

## Supplementary Material

gfae188_Supplemental_File

## Data Availability

The data underlying this article are sensitive health data and cannot be shared publicly due to privacy reasons. The data will be shared on reasonable request to the corresponding author.

## Notes

Names and affiliations of contributors are provided in the Supplemental Material
