# Supplementary material for: Remote monitoring of automated peritoneal dialysis reduces mortality, adverse events and hospitalizations: a cluster-randomized controlled trial
Source: Nephrol Dial Transplant. 2024 Aug 20;40(3):588–97. doi: 10.1093/ndt/gfae188 (PMC11997789; doi:10.1093/ndt/gfae188)

Remote monitoring of patients undergoing automated peritoneal dialysis reduces mortality, adverse events, and hospitalizations: A cluster randomized controlled trial

**Authors:**

Ramón Paniagua^1^, MD, PhD, Alfonso Ramos^2^, MD, Marcela Ávila^1^, PhD, María-de-Jesús Ventura^1^, MSC, Armando Nevares-Sida^1^, MSc, Abdul Rashid Qureshi^3^, MD, PhD, Bengt Lindholm^3^, MD, PhD, Mexican Nephrology Collaborative Study Group^4^

**SUPPLEMENTARY MATERIAL**

**1.** Complete list of Mexican Nephrology Collaborative Study Group contributors.

**2.** Clinical setting of country, institutions, and hospitals.

**3.** Training Course for Nephrologists and Nurses.

**4.** Information requested by the RM-APD device.

**5.** Suggested routines for review of data delivered by the RM-APD device.

**6.** **Table S1.** Set-up limits for flag alerts.

**7. Figure S1.** Algorithm for routine use of platform for remote patient monitoring.

**8.** Definition of Composite Indices.

**9.** Sample size calculation.

**10.** **Figure S2.** Process of recruitment of hospitals and study participants.

**11.** Additional baseline data. **Figure S3.** Age range. **Figure S4.** Range of urine volume.

**12. Table S2:** Number of hospitalizations and the incidence-rate ratio for hospitalizations in group APD versus group RM-APD.

**13. Figure S5** and **Figure S6**. Results of Cox regression analysis of Composite Index 1 and Composite Index 2 and their components.

**1. Supplemental material: List of Mexican Nephrology Collaborative Study Group contributors.**

**Steering Committee and Coordinating Center:**

Clinical monitors: Diana Pérez-Morán, Miguel A Trejo-Villeda, María D Zavaleta-Diaz, Beatriz Hernández-Franco.

Data base: Alejandro B Hinojosa-Rojas

Local investigators:

From: Hospital Central Militar (Army), Mexico City: Leysy Rosales-Chavarría, María R Romano-Bárcenas.

From: Instituto de Seguridad Social y Servicio Social de los Trabajadores del Estado (ISSSTEP), HE 5 de Mayo, Puebla, Puebla: Juana Morales-Monterrosas, Jovita Hijui-Xopa. Hospital General Darío Fernández, ISSSTE: María-Guadalupe Suárez-López.

From: Secretaría de Salud, Cd. México: HE. Belisario Domínguez, Mario Rojas-Díaz, Nancy Ávila-Ortega.

From: Instituto Mexicano del Seguro Social (IMSS) (18 hospitals):

Baja California, Mexicali, HE 30, Heriberto Reyes; Cd. México, HGR 1, Carlos McGregor, Angelica Cruz-Baltazar, Rubén Acosta-Jurado; HGZ 1A, María Begonia-Ilabaca, Patricia Gómez-Torres; HGZ 32, Emilia Cantoral-Farfán, Norberto Ávila-Osorio, Cristina Rodríguez-Esquivel, Lucina Hernández-Cervantes; HGZ 47, Fabiola Reyes, Clara V Ramírez-Loera; HGZ 8, Maritoña Camarillo, Alejandro Sánchez-Mendoza, Israel Chávez-Palacios; HGZ 2-A, Troncoso, Adrián Ramírez-Cárdenas, Ofelia Galván-Vela; Chihuahua, Chihuahua, HGR 66, Diana P García-Velásquez, Edith M De-León-Lagunas, Ofelia Sáenz-Flores; Edo. De Mex., Metepec, HGR 251, David Utrera-Ruiz, María I Rivera-Juárez; Naucalpan, HGZ 194, María A Soto-Gómez, Margarita Jiménez-Garzón; HGZ 58, Marco A Nepomuceno De Florencio, José F Álvarez-Reséndiz, Sandra Rodríguez-Badillo; Guanajuato, León, UMAE, María E Solís-Gómez, María E Reyes-López-León; Jalisco, Guadalajara, HGR 46, María L Romo-Flores, Víctor I Tejeda-González; HGZ 45, Laura E Aguilar-Fletes, Samara A Plascencia-Coutiño; HGZ 89, Ámbar P Uriarte-Loaiza, Martha A Padilla-Mercado, Karina Arroyo-Cuevas; Tlajomulco, HGR 180, Laura M Díaz-Canchola, Laura Quezada-Jauregui; Michoacán, Morelia, UMAA 75, Daniel Gil-Romero, Alma D Cansino-Villagómez; Nuevo León, Monterrey, HGZ 33, Silverio Lara-Robles, María T Muñoz-Rivera; HGZ 6, Rosenda A Zurita-Rodríguez, Teresita J Rodríguez-Vega.

*Abbreviations:* HE: Hospital de Especialidades. HGR: Hospital General Regional; HGZ: Hospital General de Zona. UMAA: Unidad de Medicina Ambulatoria. UMAE: Unidad Médica de Alta Especialidad.

**2.** **Supplemental material: Clinical setting of the country, institution, and hospital network where PD patients are attended.**

Mexico has >126 million inhabitants, the median age is 29 years, 6.3 million are older than 65 years, 46 million are in condition of poverty, and 4.9% have less than elemental education.

PD is provided by the Instituto Mexicano del Seguro Social (IMSS) as a part of the Health Insurance. Information is available in:

Informe al Ejecutivo Federal y al Congreso de la Unión sobre la Situación Financiera y los Riesgos del Instituto Mexicano del Seguro Social 2022-2023. **[Report to the Federal Executive and the Congress of the Union on the Financial Situation and Risks of the Mexican Social Security Institute 2022-2023]**. www.imss.gob.mx/conoce-al-imss/informes-estadisticas

The population covered by the Health Insurance is 61,141,849 inhabitants. Two hundred and forty-nine hospitals (Second and third levels) have PD and HD programs. The population that receives dialysis (PD+HD) as of February 2024 was 79,605 patients. Of them 53.97% receive HD and 46.03% PD. From the last group, 40.94% receive APD and 59.06% receive CAPD. (Data available in <http://cpim.imss.gob.mx>. Consulted in May 2024). The average number of PD patients per hospital is 149.

The assignment to each modality is decided by the Renal Replacement Therapies Committee of each hospital. The members of the committee are: The director of the hospital, the Chief of Nephrology, at least one Nephrologist from the staff, one Psychiatrist or Psychologist, the Chief of Nurses, Chief of Nephrology Nurses, the Nutritionist, and the Social Worker. Described in:

Procedimiento para otorgar el tratamiento dialítico de los pacientes con Insuficiencia Renal Crónica en Unidades Médicas Hospitalarias de Segundo Nivel de Atención 2660-002-057 **[Procedure to grant dialytic treatment to patients with Chronic Kidney Failure in Second Level Hospital Medical Units. 2660-002-057]**. http://reposipot.imss.gob.mx › Procedimiento 2660-002-057

Hospitals from the Army and ISSSTEP were individually included; however, they have similar organizational structures and functions.

**Requirements for hospital inclusion.**

- Hospitals with established (> 5 years) peritoneal dialysis programs with both modalities, automated and continuous ambulatory.
- More than 100 prevalent patients in the program, and 50 new patients by year.
- Nephrologists and nurses trained in PD assigned to the program.
- Nutritionists and social workers assigned to the program.
- Physical area for catheter placement.
- Room for training patients and caregivers in PD; for beginners, and those who need retraining.
- Emergency room
- Radiology department.
- Laboratory for biochemical and microbiological analysis.
- Surgery, cardiology, and infection consultants.
- Director providing informed consent for participation in the study.

**3. Supplemental material: Training Course for Nephrologists and Nurses (First Course, before enrollment of patients).**

Day 1. Part 1.

- Critical data for patient PD monitoring and needs for change prescriptions.
- Screen messages during installation of the device, at turning-on, sending information from device to cloud.
- Setting up yellow and red flags, rationale for recommended limits.
- Structure of Sharesource reports and formats.
- Interpreting information delivered from device.
- Graphical reports inflow and outflow of dialysis solution patterns.

Day 1. Part 2.

- Non-compliance.
- Low drain volumes, alarms, bypass, residual volume, wet day, initial drainage.
- Patterns. Slow inflow-outflow, fibrin, air.
- Early end.
- Ultrafiltration.

Day 2. Part 1.

- Practice of algorithms of reports review
- Analyses and discussion of specific problems. Compliance

Day 2, Part 2.

Interpreting flags and implementing solutions in:

Treatment duration Lost treatment time

Treatment variations Lost Dwell Time

Lost therapy volume

Drain finished early

Initial drain variation

High drain volume

Interruption or changes in inflow-outflow patterns

Patient intervention Bypass count in infusion or dwell

System alerts Events during treatment

**4. Supplemental material: Information requested by the device.**

The equipment requests input information about the type of solution infused, the daily body weight (with the peritoneal cavity empty) and blood pressure at the beginning of each session as it is mentioned in the Supplemental material.

**5. Supplemental material: Suggested routines for review of data delivered from RM-APD**

Nurses and nephrologists from all hospitals were suggested to review the initial screen (Panoramic more recent seven consecutive days) view of the data report, looking for red and yellow flags daily. They must decide if remote actions should be performed. Among the necessary actions were a phone call if the missing day of connection is repeated (urgent if two or more), early finish of drainage, or stop of session, three or more times of lost dwell time, loss of prescribed volume of PD solution, initial drain variation, changes in drain volume, bypass count in infusion or dwell, number of events during treatment. The points set are shown in Table S1 below, and the flow chart with the algorithm for routine analysis for the suggested daily review is in Figure S1.

**6. Supplemental material. Table S1. Suggested set-up limits for flag alerts.**

| **Table S1. Set-up limits for flag alerts** | | | |
| --- | --- | --- | --- |
|  |  | Yellow flag | Red flag |
| Treatment duration | Lost treatment time | 15 min | 30 min |
| Treatment variations | Lost dwell time | 15 min | 30 min |
|  | Lost therapy volume | 5% | 10% |
|  | Drain finished early |  | 2 |
|  | Initial drain variation | 50% | 100% |
|  | High drain volume |  | X |
| Patient intervention | Bypass count in infusion or dwell |  | 2 |
| System alerts | Events during treatment | 5 | 10 |

**7.Supplement material.**

**Figure S1: Algorithm for routine use of remote patient monitoring.**

**
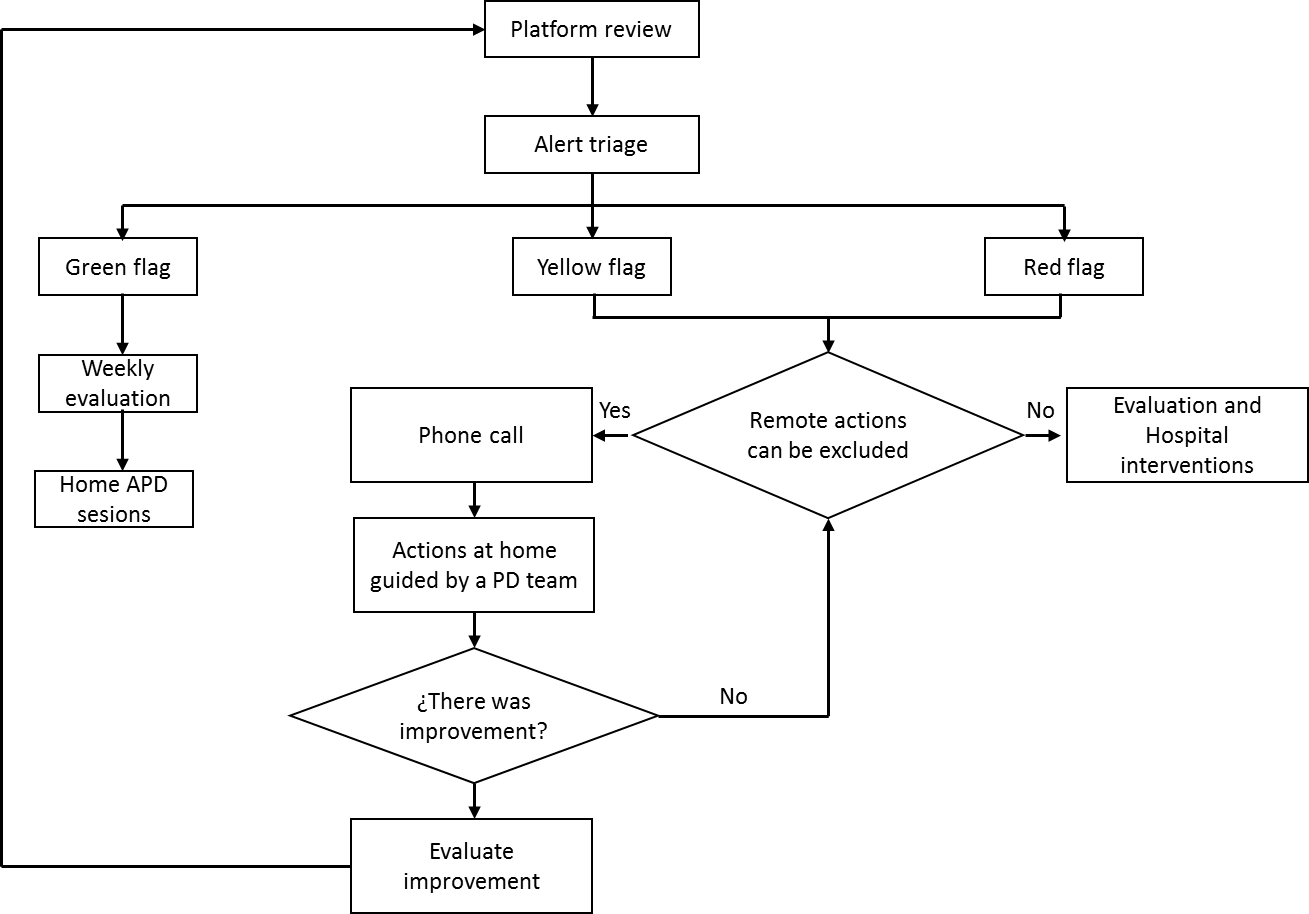
**

**Comment:** Routines may appear as time-consuming; despite that, the new routine was well-accepted by doctors and nurses, emphasizing that they needed to become familiarized with the process, and after that the review becomes easier and faster.

**8. Supplemental material:**

**Definition of Composite Indices**

- Composite Index 1 (CI 1) ultrafiltration-associated events
- Heart Failure: New York Heart Association Functional Classification.
- Cardiovascular Dysautonomia: include reflex syncope, inappropriate sinus tachycardia, and syndromes of orthostatic intolerance: orthostatic hypotension (OH) and postural orthostatic tachycardia syndrome (POTS).
- Acute Myocardial Infarct: Electrocardiographic signs.
- Syncope: Attested transient loss of consciousness
- Edema: Abnormally swollen with fluid in feet, ankles and legs of at least Grade 1. (Classified as: Grade 1: The pressure leaves an indentation of 3–4 mm that rebounds in fewer than 15 seconds. Grade 2: The pressure leaves an indentation of 3–4 mm that rebounds in more than 15 seconds)
- Stroke: Extracranial computed tomography (CT) signs.
- Pleural effusion: Radiographic signs.
- Angina: A pressing, squeezing, or crushing pain, usually in the chest under your breastbone, radiating in arms, shoulders, jaw, neck, or back, shortness of breath, weakness and fatigue, feeling faint.
- Hypertensive Crisis: Sudden, severe increase in blood pressure, pressure reading of 180/120 millimeters of mercury (mm Hg) or greater.
- Sudden death: Unexpected death that is instantaneous or occurs within minutes from any cause other than violence.

**Events that could be associated with ineffective dialysis.**

Uremia: Manifestations, such as pruritus, anorexia, nausea, vomiting, fatigue, weight loss, muscle cramps, mental status changes.

Malnutrition: Involuntary weight loss of 10% or more 6 months, or 5% or more in 1 month. Albumin: <3.5g/dL,

Acidosis: total venous bicarbonate <22 mM.

Hyperkalemia: Serum potassium >5.5mM/L

**9. Supplemental material. Sample size calculation.**

To calculate sample size, we used unpublished data from our previous study of an institutional multicenter cohort with 457 incident patients on PD showing a rate of 15 events / 100 patient years when using a composite index, which like the one applied in the present study included the combination of mortality, hospitalizations, potentially preventable hospitalizations, and unplanned visits. We applied this rate for the control group (APD without remote monitoring device) and assumed a rate of 8 events / 100 patient years in the intervention group (APD with RM telemedicine device). A power of 0.80 was set, with α of 0.05, and k (intraclass correlation coefficient, ICC) of 0.03. For 7 clusters per group, the calculated sample is 51 patients for each cluster (Donner A, Klar N. Design and Analysis of Cluster Randomization; Trials in Health Research: Arnold, London 2000., Hayes RJ, Moulton LH. Cluster Randomized Trials. Chapman and Hall/CRC. 2017; 424).

**10. Supplemental material: Supplement Figure S2.** Schematic flow chart of the process of recruitment of hospitals and study participants.
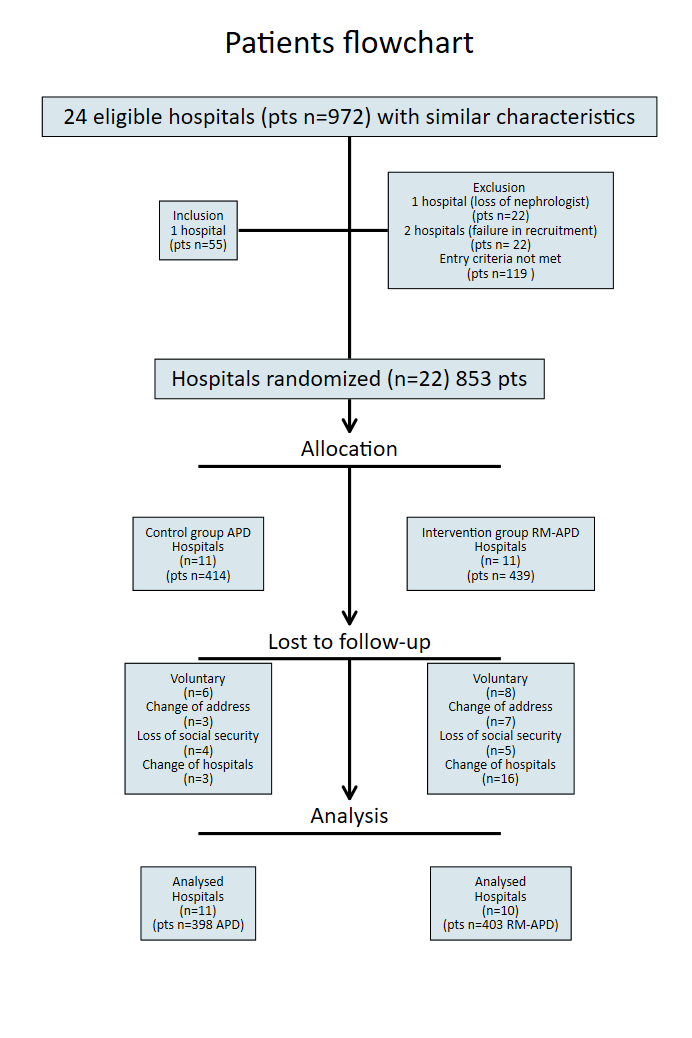


**11. Supplemental material. Additional Baseline data:** Age range (Figure S3), Range of urine volume (Figure S4), Additional information about baseline variables

**Supplement Figure S3.** Age range.

Comment: Age tended to be different between the two groups but the difference did not reach statistical significance, and the distribution was similar in the two groups.

**Supplement Figure S4. Range of urine volume.**

**
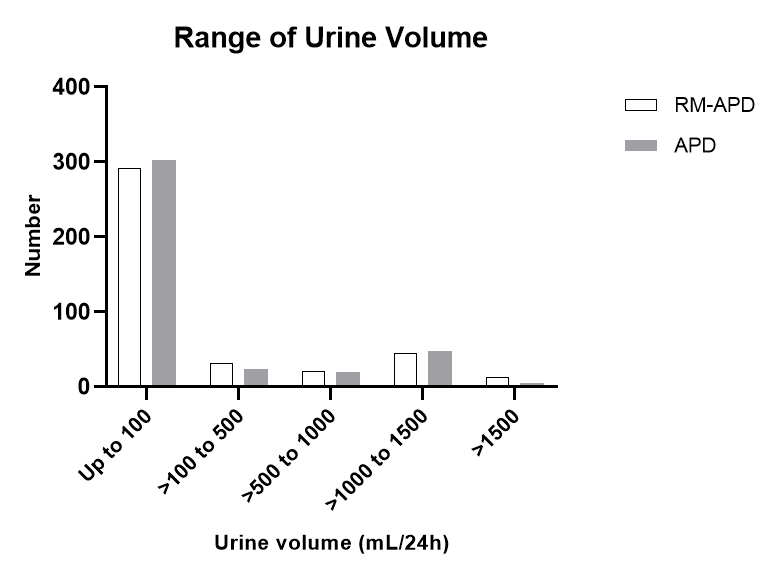
**

Comment: The number of patients with significant urinary volumes (≥100 mL/24h) was similar in the two groups, see distribution in figure above. The average urine volume, 840±354 ml in APD and 947±535 ml in RM-APD (p= 0.095) tended to be higher in the RM-APD group but this difference did not reach statistical significance.

**Additional information about baseline variables with differences close to significance between groups.**

- The hypertension data corresponds to the presence of hypertension prior to the onset of PD and was collected from the clinical history; however, it cannot be stated as a cause of CKD or its persistence throughout follow-up.
- Peripheral vascular disease was more frequent in APD group; after recognition of differences at baseline, the data were carefully reviewed and showed that all patients reached the end of the study alive and well. Therefore, we conclude that the presence of peripheral vascular disease had no effect on survival.

**12. Supplemental material. Table S2: Number of hospitalizations and the incidence-rate ratio for hospitalizations in group APD versus group RM-APD**

**Supplement Table S2.** Number of hospitalizations and the incidence-rate ratio for hospitalizations in group APD versus group RM-APD: Number of adverse events and the incidence-rate ratio (IRR) for hospitalizations due to different reasons (mechanical, fluid overload (FOL), insufficient dialysis) and combination of reasons in group APD versus group RM-APD, adjusting for patients’ identification and centers.

| Reasons for hospitalization | Crude APD /RM-APD | IRR (95% CI) | P>\|z\| |
| --- | --- | --- | --- |
| **Mechanical Hospitalization events** | **18/45 (p=0.004)** | **0.41 (0.22-0.66)** | **0.001** |
| Mech and Infection Hospitalization events | 0/2 | NA | NA |
| Mechanical and FOL Hospitalizations | 2/1 | NA | NA |
| Mech and Insufficient Dialysis Hospitalization | **0/4** | NA | NA |
| Infection and FOL Hospitalization | 6/4 | NA | NA |
| Infection and Insufficient Dialysis Hospitalization | 5/4 | NA | NA |
| **FOL and Insufficient Dialysis Hospitalization** | **19/7 (p=0.01)** | **3.24 (1.24-7.53)** | **0.03** |

Results of crude analysis are calculated with Chi-square test. IRR=Incidence-rate ratio is calculated with zero-inflated Poisson model with Bayesian method.

**13. Supplement Figure S5.** Cumulative incidence rates of Composite Index-1 and its three components, all-cause mortality, adverse events, and hospitalization of any cause for patients receiving APD and RM-APD. Inserts show results of Cox regression analysis with hazard ratio (HR) and 95% confidence intervals (95%CI) for these outcomes in patients using APD and RM-APD respectively after adjusting for age, sex, and center.


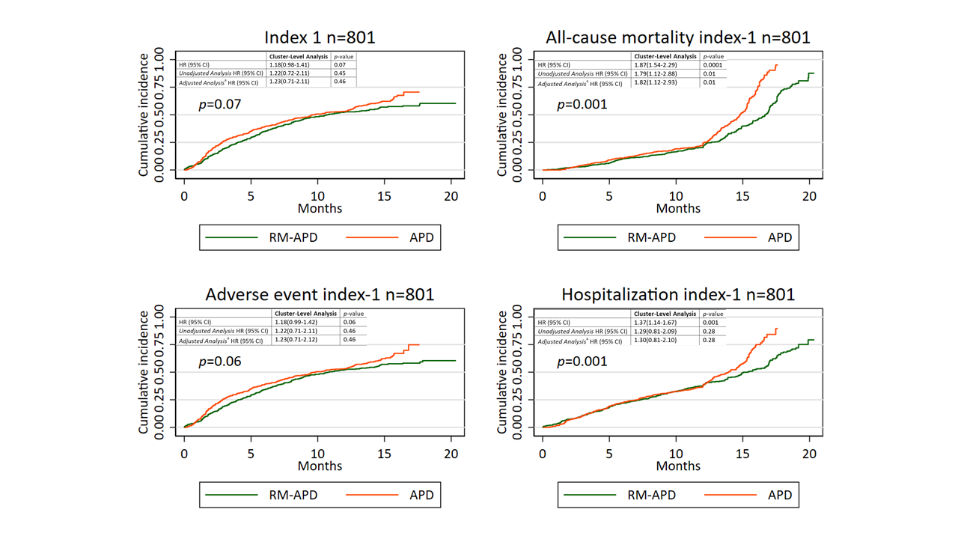


**Supplement Figure S6.** Cumulative incidence rates of Composite Index 2 and its three components, cardiovascular mortality, and adverse events and hospitalization of cardiovascular origin or related to fluid overload or inefficient dialysis. Inserts show results of Cox regression analysis with hazard ratio (HR) and 95% confidence intervals (95%CI) for these outcomes in patients using APD and RM-APD respectively after adjusting for age, sex and center.


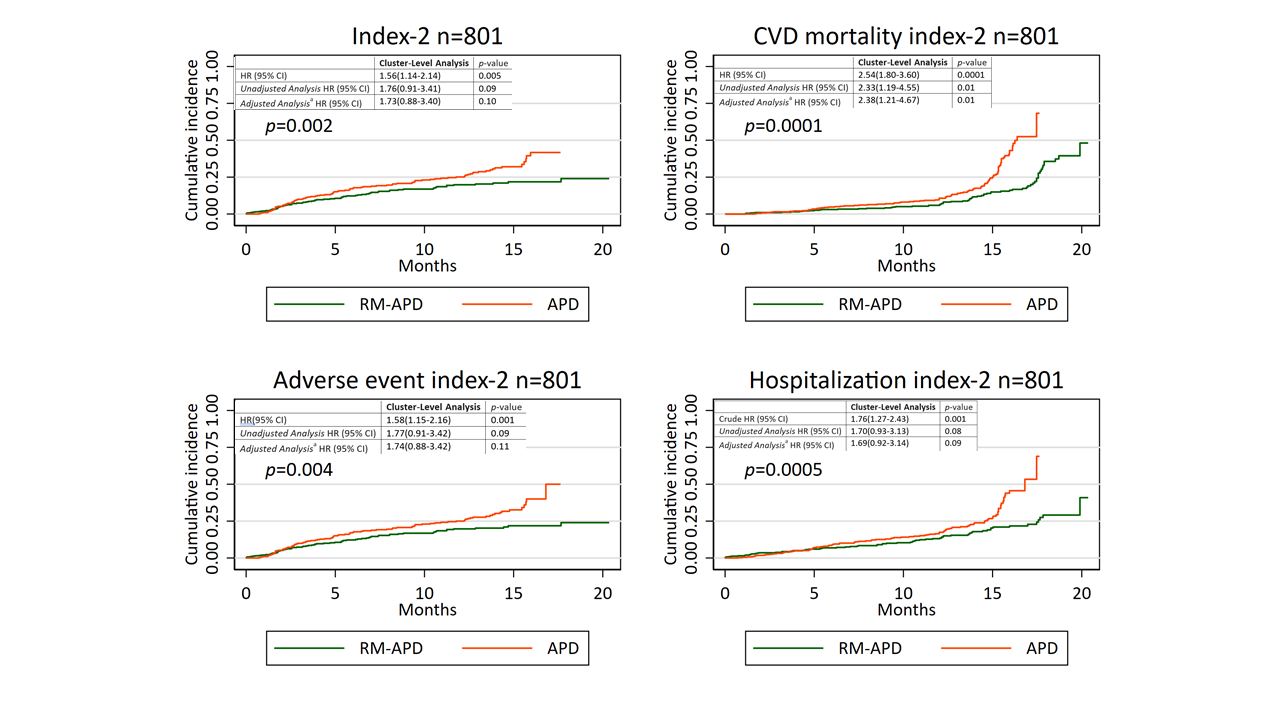

Supplement: gfae188_Supplemental_File [file gfae188_Supplemental_File.docx]
